# Urinary Biomarkers of Prenatal Atrazine Exposure and Adverse Birth Outcomes in the PELAGIE Birth Cohort

**DOI:** 10.1289/ehp.1002775

**Published:** 2011-03-02

**Authors:** Cécile Chevrier, Gwendolina Limon, Christine Monfort, Florence Rouget, Ronan Garlantézec, Claire Petit, Gaël Durand, Sylvaine Cordier

**Affiliations:** 1 INSERM, U625, Rennes, France; 2 University of Rennes I, IFR140, Rennes, France; 3 Idhesa, Plouzané, France; 4 ”Bien naître en Ille-et-Vilaine” Perinatal Network, Rennes, France; 5 Public Health Department, Hospital University, Brest, France

**Keywords:** atrazine, environmental exposure, fetal growth, herbicides

## Abstract

Background: Despite evidence of atrazine toxicity in developing organisms from experimental studies, few studies—and fewer epidemiologic investigations—have examined the potential effects of prenatal exposure.

Objectives: We assessed the association between adverse birth outcomes and urinary biomarkers of prenatal atrazine exposure, while taking into account exposures to other herbicides used on corn crops (simazine, alachlor, metolachlor, and acetochlor).

Methods: This study used a case-cohort design nested in a prospective birth cohort conducted in the Brittany region of France from 2002 through 2006. We collected maternal urine samples to examine pesticide exposure biomarkers before the 19th week of gestation.

Results: We found quantifiable levels of atrazine or atrazine mercapturate in urine samples from 5.5% of 579 pregnant women, and dealkylated and identified hydroxylated triazine metabolites in 20% and 40% of samples, respectively. The presence versus absence of quantifiable levels of atrazine or a specific atrazine metabolite was associated with fetal growth restriction [odds ratio (OR) = 1.5; 95% confidence interval (CI), 1.0–2.2] and small head circumference for sex and gestational age (OR = 1.7; 95% CI, 1.0–2.7). Associations with major congenital anomalies were not evident with atrazine or its specific metabolites. Head circumference was inversely associated with the presence of quantifiable urinary metolachlor.

Conclusions: This study is the first to assess associations of birth outcomes with multiple urinary biomarkers of exposure to triazine and chloroacetanilide herbicides. Evidence of associations with adverse birth outcomes raises particular concerns for countries where atrazine is still in use.

Atrazine is a triazine herbicide used to control broadleaf and grassy weeds in crops, mainly corn and sorghum. Although the European Union banned atrazine in 2001 because it is a ubiquitous water contaminant, it is still used in > 70 countries, including the United States, Brazil, Argentina, Mexico, and China (LeBaron et al. 2008). In 2001, agricultural pesticide use in France was among the highest in Europe, averaging 3.4 kg/hectare of agricultural area (Benoît et al. 2005). The intensive regional agricultural activities from pig, poultry, and dairy farming in the Brittany region (northwestern France) make pesticide exposure a critical issue. Corn crops, especially animal feed corn, are common there. In 2001, experts estimated that 200 tons of atrazine was applied to 70% of the corn grown in Brittany, for an average of 0.620 kg of atrazine per hectare (Agreste Bretagne 2001). In 1997, the Ministry of Agriculture limited atrazine to 1 kg/ha/year; in 2002, it banned the sale of numerous products containing atrazine and, in October 2003, finally banned atrazine use. The primary replacements have been alachlor and acetochlor, both chloroacetanilide herbicides.

Once released into the environment, atrazine is transformed over time by various chemical, photochemical, and biologically mediated reactions into other compounds, called degradates or metabolites. Water contamination by atrazine and its degradates, including hydroxyatrazine and desethylatrazine, has decreased in Brittany since 2003, but many surveys still show environmental contamination in the region. Testing indicates that 27% of water samples from Breton rivers had atrazine levels > 0.1 μg/L in 2003 compared with 10% of samples in 2004, and only 1% in 2007, whereas atrazine degradates were detected in 76% of water samples in 2003 and 20% in 2007 [Réseau CORPEP (Cellule d’Orientation Régionale pour la Protection des Eaux contre les Pesticides) 2008]. Atrazine residues were also detected in surface and underground sources of human drinking water (Marchand 2006). In 2006 the maximum measured surface water concentration was 0.38 μg/L for atrazine and 0.37 μg/L for hydroxyatrazine, and underground levels, 0.14 μg/L for atrazine, 0.38 μg/L for hydroxyatrazine, and 0.32 μg/L for desethylatrazine. Finally, in drinking water of Brittany in 2005, when the regulatory limits of pesticides (0.1 μg/L) were exceeded, it was mainly due to desethylatrazine (67%) and atrazine (17%) residues (Marchand 2006).

Some epidemiologic studies have looked at the potential impact of prenatal exposure to atrazine or its environmental degradates on pregnancy outcomes in the general population. Munger et al. (1997) collected data from 1984 through 1990 and observed higher rates of babies born small for gestational age (SGA) in Iowa (USA) communities with higher atrazine levels in their drinking water. In Brittany, Villanueva et al. (2005) found that the risk of SGA increased with the number of months of the third trimester of pregnancy occurring from May to September, months with the highest atrazine levels in drinking water there from 1990 through 1998. Ochoa-Acuña et al. (2009) found a statistically significant increase in SGA prevalence associated with atrazine in drinking water in Indiana (USA) from 1993 through 2007. In Washington State (USA), Waller et al. (2010) observed a higher risk of gastroschisis from 2001 through 2006 among babies of mothers who lived closer to sites with high atrazine concentrations. Three other studies related high incidences of birth defects to counties with high triazine use in New York (USA) from 1995 through 1999 (Davis 2005), to the highest mean atrazine levels in surface water in Indiana from 1990 through 2002 (Mattix et al. 2007), and to the annual April–July peak of atrazine contamination of surface water [based on 1996–2002 U.S. data (Winchester et al. 2009)]. Most of these studies were retrospective and relied on ecological exposure assessment. Co-occurring chemicals make it impossible to determine whether associations are specific to atrazine exposure in the general population.

Our aim was to assess the association between adverse birth outcomes and urinary biomarkers of prenatal atrazine exposure, taking into account other herbicides potentially used on corn, including simazine, alachlor, metolachlor, and acetochlor. This study is based on a prospective population-based cohort in the Brittany region—the PELAGIE (Perturbateurs endocriniens: Étude Longitudinale sur les Anomalies de la Grossesse, l’Infertilité et l’Enfance) cohort—that collected urine samples from pregnant women between 2002 and 2006 to assess pesticide exposure biomarkers before the 19th week of gestation.

## Materials and Methods

*The PELAGIE cohort.* The PELAGIE cohort included 3,421 pregnant women from Brittany from 2002 through February 2006. Enrollment started in the district of Ille-et-Vilaine, continued in Finistère, and ended in Côtes d’Armor. Gynecologists, obstetricians, and ultrasonographers recruited women during consultations in early pregnancy, informing them about the nature of the study and asking them to participate (after providing written consent). Women were enrolled before the 19th week of gestation after providing written informed consent and completing a questionnaire at home concerning family social and demographic characteristics, diet, and lifestyle. Women were asked to return the questionnaire by mail, along with a first-morning-void urine sample that they collected and transferred into two vials containing nitric acid (to inhibit bacterial proliferation). Samples were mailed to the study laboratory (INSERM U625, Rennes, France) in a prestamped package at ambient temperature, with routine delivery taking from 1 to 3 days. On receipt, samples were frozen and stored at –20°C. Midwives and pediatricians at the maternity units provided study staff with medical information about the pregnancy, delivery, and neonatal health for 3,399 women (99.4% of the cohort; the other 22 women were lost to follow-up). Birth weight, length, and head circumference were obtained from hospital records by pediatricians. Gestational age was based on both date of the last menstrual period and ultrasound examinations. The appropriate national ethics committees approved the study.

*Outcome definition.* Live-born singleton children of the cohort without major congenital anomalies were classified as fetal growth restricted (FGR) if their birth weight was below the 5th percentile of the distribution of expected birth weight modeled within the cohort according to gestational age (third-degree polynomial), sex, parity (third-degree polynomial), maternal weight (third-degree polynomial), height, and age (second-degree polynomial) (Mamelle et al. 2001). Small head circumference (SHC) was defined by a head circumference below the 5th percentile of the birth head circumference distribution for a given gestational age and sex, according to French reference curves (Mamelle et al. 1996). Major congenital malformations in live-born infants were diagnosed by hospital staff pediatricians at the maternity unit, based on findings at the clinical examination at birth. In addition, pediatricians were asked specifically about male genital anomalies (hypospadias, undescended testis, and micropenis). Diagnosis validation by pediatric surgeons was obtained for half of these male genital anomalies (*n* = 12 of 26) up to 2 years after birth. For detailed information on the classification of congenital malformations in this cohort, see Garlantézec et al. (2009).

*Data on corn agricultural activities and atrazine water contamination.* Data on agricultural activities were collected for each mother’s municipality of residence at enrollment from the national agricultural census conducted in 2000; specifically, we recorded the proportion of the municipality’s area used for corn crops. Drinking water contamination by atrazine was routinely assessed until its year of ban by the office of Social and Sanitary Affairs of Brittany (Rennes, France). We collected these data from 2000 to 2002 for municipalities in Ille-et-Vilaine and used them to estimate the average atrazine level in water during the first trimester of pregnancy for participants residing in these locations. Exposure to atrazine via tap water was then estimated by multiplying the average concentrations by the tap water consumption reported in the questionnaire.

*Case-cohort design.* The present study was based on a case-cohort design (Kass and Gold 2004) that included 601 children randomly selected from the entire live-born singleton cohort (18%), referred to as the subcohort, and groups with adverse birth outcomes, including congenital anomalies (88 major anomalies, 26 male genital anomalies), FGR (*n* = 180), and SHC (*n* = 105), representing all of the cohort members with these outcomes.

*Pesticide determination in urine samples.* Pesticides were measured in urine samples from 579 women in the subcohort (96%), all mothers of babies with major congenital anomalies or male genital anomalies, 178 mothers of children with FGR, and 103 mothers with children with SHC. First-morning-void urine samples, which are very likely to contain recent traces of exposure to the nonpersistent pesticides we targeted (Barr et al. 2005), were used to determine the presence of 12 triazine compounds: atrazine and its glutathione-derived metabolite atrazine mercapturate, simazine and its metabolite simazine mercapturate, three dealkylated triazine metabolites that are likely to be formed in water systems (desethylatrazine, desisopropyl atrazine, and 2-chlorodiaminoatrazine), and five hydroxylated triazine metabolites, mostly formed in plants [hydroxyatrazine, hydroxysimazine, hydroxydesethylatrazine, hydroxy-desisopropyl atrazine, and hydroxy-2-chlorodiaminoatrazine (ammeline)]. The chloroacetanilide herbicides alachlor, metolachlor, and acetolachlor were also measured, together with 2,6-diethylaniline, an alachlor metabolite.

Chemical analyses were performed by the Idhesa Institute (Plouzané, France) on urine samples (maximum, 10 mL) with liquid chromatography/triple-quadrupole mass spectrometry (LC/MS-MS) after solid-phase extraction (SPE). Triazine and chloroacetanilide herbicides were analyzed simultaneously. Reference standards for pesticides and their metabolites were purchased from the Ehrenstörfer laboratory (Augsburg, Germany) and from Promochem (Teddington, UK). Other purchased chemicals included LC/MS-grade acetonitrile and methanol from Fisher Scientific (> 99%; Loughborough, Leicestershire, UK), analytical-grade formic acid from Baker (98%; Deventer, Netherlands), and gas from Air Liquide (> 99%; Paris, France). Standard solutions were prepared in methanol or acetonitrile and stored at –20°C. Simazine-d10 was used as internal standard for extraction and detection controls. Calibration standards were prepared by adding appropriate working standard solutions to 10-mL fresh samples of pesticide-free human urine before extraction to obtain concentrations in the calibration range. Isotope-labeled standards and surrogates were added to calibration standards to obtain a final concentration of 1 μg/L. The urine samples were thawed and shaken. Preconcentration and extraction (5 mL) were performed with an online SPE high-volume symbiosis system (Spark Holland, Emmen, Netherlands)and the Hysphere C18 HD cartridge (2 × 10 mm; Spark Holland), and analytes were eluted during the mobile phase. Separation was performed with Synergy fusion-RP analytical column (250 mm × 2.0 mm, 4 μm particle diameter; Phenomenex, Torrance, CA, USA) preceded by a guard column (4 mm × 2 mm) of the same packing material from Phenomenex. The mobile phase was a gradient of a mixture of 5 mm ammonium formate/formic acid 0.01% and acetonitrile/formic acid 0.01%. The flow rate was 0.2 mL/min, and the temperature 35°C. LC/MS-MS analyses were performed with a system composed of a Waters alliance 2690 LC pump equipped with an autosampler and connected in series with a Quattro Ultima triple-quadrupole mass spectrometer (Waters-Micromass, Manchester, UK), equipped for electrospray ionization. Acquisition took place in multiple-reaction monitoring mode, with two transitions per compound (one for quantification and one for confirmation) in positive ionization mode. All validation procedures were performed with fresh samples of herbicide-free human urine. The limit of detection (LOD) was defined as a signal three times the background noise at the lowest analyte concentration assayed, and the limit of quantification (LOQ) as 10 times the background noise. LOQs ranged from 0.001 to 1.7 μg/L. Concentration ranges were linear from 0.010 to 10 μg/L for herbicides with the lowest LODs (metolachlor, desethylatrazine, alachlor, 2,6-diethylaniline) and were linear from the LOD for the others. Average recoveries were 100% ± 20%, with coefficients of variation ranging from 0.1% to 13.9%. Stability tests showed, among doped human urinary samples, a slight decrease in levels after 32 hr at ambient temperatures (respectively, –7% and –9% for atrazine and atrazine mercapturate) and no changes according to the presence of nitric acid. This method is reported to provide minimal sample handling, good extraction recovery, and satisfactory LODs and LOQs (Panuwet et al. 2008, 2010).

*Definition of exposure profiles from urinary concentrations.* Most human biomonitoring studies assess atrazine exposure by measuring atrazine mercapturate in urine samples. This metabolite is formed directly from atrazine and cannot result from other atrazine degradation processes in the environment or organisms (see Barr et al. 2007, their Figure 1). However, measurement of this metabolite alone misrepresents total atrazine exposure (Barr et al. 2007). In our study, mothers were considered exposed to atrazine when atrazine or at least one of its specific metabolites (i.e., formed only from atrazine degradation processes: atrazine mercapturate, desethylatrazine, hydroxyatrazine, or hydroxydesethylatrazine) was quantified in their urine. Among them, mothers with quantifiable levels of atrazine or atrazine mercapturate were classified as “directly exposed” to atrazine. We classified mothers as unexposed if there was no trace (i.e., < LOQ) of atrazine or any of its four specific metabolites in their urine sample. We used a similar strategy to classify exposure to simazine and its specific metabolites. We also classified women as exposed to dealkylated or hydroxylated triazine metabolites if at least one metabolite in the respective group was quantified in the urine sample, and as exposed to alachlor if alachlor or its metabolite 2,6-diethylaniline was quantified. Molar concentrations (nanomoles per liter) were summed to compute combined biomarker levels for groups of related compounds. For this computation, nonquantified values were not imputed (or were set at zero), and molar concentrations were computed by dividing the chemical determination value, expressed in micrograms per liter, by the molar mass.

**Figure 1:**
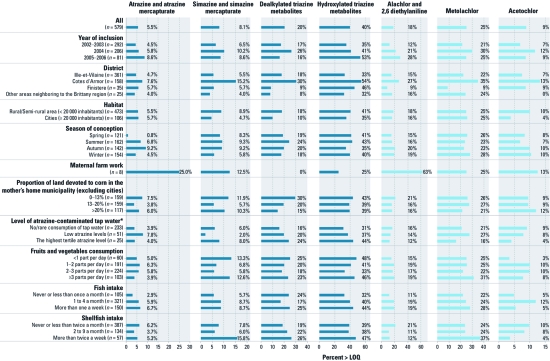
Possible determinants of triazine and chloroacetanilide herbicide exposure shown by urinary biomarkers in 579 pregnant women randomly selected from the PELAGIE cohort (France, 2002-2006). Dealkylated triazine metabolites are desethylatrazine, desisopropyl atrazine, and 2-chlorodiaminoatrazine; hydroxylated triazine metabolites are hydroxyatrazine, hydroxysimazine, hydroxydesethylatrazine, hydroxy desisopropyl atrazine, and ammeline. *Data available only for the Ille-et-Vilaine district.

*Statistical analyses.* Logistic models were used to estimate associations between biomarkers and adverse birth outcomes. We did not incorporate case-cohort sampling probabilities because the case-cohort odds ratio (OR) approximates the case–control OR when the outcome is rare (Kass and Gold 2004). Covariates considered in multivariate analyses were known or suspected risk factors and factors that predicted outcomes or urinary concentrations in this cohort. An initial list of covariates, used for the FGR models, included year of enrollment (2002–2003, 2004, 2005–2006), residence district (Ille-et-Vilaine, Côtes d’Armor, Finistère, or other), maternal age (< 25, 25–30, 30–35, > 35 years), education level (primary/secondary school, baccalaureate, technical school/postgraduate), smoking status at enrollment (nonsmoker, nonsmoker who smoked at conception, smoker), alcohol consumption at enrollment (abstinent, occasional, ≥ 1 drink per day), high blood pressure before/during pregnancy, season at conception, thawing and refreezing process (a subset of 52 samples), and urine creatinine level (milligrams per liter). For SHC models, we also considered cesarean delivery (because passage through the birth canal may influence head circumference at birth), parity (1, 2, ≥ 3), and prepregnancy body mass index (BMI; < 18.5, 18.5–25, 25–30, ≥ 30 kg/m^2^). For congenital anomalies models, in addition to the initial list, we considered folic acid intake during the 3 months before enrollment (through vitamin supplementation), maternal fever in early pregnancy (before enrollment), child’s sex, parity, prepregnancy BMI, mother’s occupational exposure to solvents (no, yes, no job) [in accordance with previous associations observed from this cohort (Garlantézec et al. 2009)], and gestational age at birth (in weeks). Linear models were used to estimate associations between biomarkers and birth weight, birth length, and head circumference as continuous outcomes, using the same initial list of covariates plus child’s sex, parity, prepregnancy BMI, gestational age at birth, and squared gestational age, as well as fish intake (three categories) and shellfish intake (three categories) according to previous results from this cohort (Guldner et al. 2007) and, for head circumference, cesarean delivery. For all outcomes, the list of covariates also included exposures to herbicides, as categorical variables as defined above.

Final models were obtained using a backward selection process of the covariates (*p* < 0.20) and forcing in models the exposure of interest. Additional analyses considered biomarker levels as continuous in models and were thus restricted to samples with quantifiable levels of the compound of interest. SAS software was used for all calculations (SAS/STAT version 9.1; SAS Institute Inc., Cary, NC, USA).

## Results

*Population.* Table 1 describes the case-cohort population. We recruited most women in the random subcohort in Ille-et-Vilaine during 2003 and 2004. Brittany’s population density is low, and 18% of the women lived in cities (> 20,000 inhabitants). Average maternal age was 30 years, and most were postgraduates. Few (*n* = 8) were farmers. At enrollment, mean gestational age was 11.7 weeks, 16% of mothers smoked, and 15% reported drinking alcohol occasionally or regularly. Prepregnancy BMI was 18.5–25 kg/m^2^ (normal range) for 75%. Five percent had high blood pressure during pregnancy, and 17% of deliveries were by cesarean.

**Table 1:** Characteristics of the case-cohort population of pregnant women assessed for urinary biomarkers of pesticide exposure (France, 2002–2006).

Table 1. Characteristics of the case-cohort population of pregnant women assessed for urinary biomarkers of pesticide exposure (France, 2002–2006).													
	Subcohort			Major congenital anomalies			FGR			SHC			Mean ± SE (minimum–maximum)
Characteristic	*n *(%)			*n *(%)			*n *(%)			*n *(%)			
All	579			88			178			103			
Year of inclusion													
2002–2003	292	(50)		51	(58)		89	(50)		43	(42)		
2004	206	(36)		23	(26)		60	(34)		48	(47)		
2005–2006	81	(14)		14	(16)		29	(16)		12	(12)		
District													
Ille-et-Vilaine	361	(62)		63	(72)		112	(63)		58	(56)		
Côtes d’Armor	158	(27)		21	(24)		53	(30)		42	(41)		
Finistère	35	(6)		3	(3)		8	(4)		1	(1)		
Other	25	(4)		1	(1)		5	(3)		2	(2)		
Habitat													
Rural/semirural area (< 20,000 inhabitants)	473	(82)		72	(82)		151	(85)		92	(89)		
Urban (≥ 20,000 inhabitants)	106	(18)		16	(18)		27	(15)		11	(11)		
Maternal age (years)													30 ± 0.2 (19–44)
< 25	73	(13)		9	(10)		25	(14)		16	(15)		
25–30	224	(39)		30	(34)		60	(34)		38	(37)		
30–35	202	(35)		34	(39)		68	(38)		39	(38)		
≥ 35	80	(14)		15	(17)		25	(14)		10	(10)		
Maternal education													
Primary/secondary school	109	(19)		17	(19)		47	(26)		15	(15)		
Baccalaureate	109	(19)		16	(18)		28	(16)		24	(23)		
Technical school/Postsecondary	360	(62)		55	(63)		103	(58)		64	(62)		
Missing	1												
Maternal farmwork	8	(1.7)		1	(1.1)		3	(2)		2	(2)		
Maternal smoking status at enrollment													
Nonsmoker	411	(72)		70	(80)		118	(66)		66	(65)		
Nonsmoker but smoked at conception	70	(12)		9	(10)		17	(10)		13	(13)		
Smoker	91	(16)		9	(10)		43	(24)		23	(22)		
Missing	7									1			
Maternal drinking status at enrollment													
Abstinent	486	(85)		77	(89)		149	(85)		89	(89)		
Occasional (< 1 drink per day)	75	(13)		9	(10)		19	(11)		8	(8)		
≥ 1 drink per day	9	(2)		1	(1)		7	(4)		3	(3)		
Missing	9			1			3			3			
Season of conception													
Spring	121	(21)		23	(26)		35	(20)		23	(22)		
Summer	162	(28)		15	(17)		48	(27)		34	(33)		
Autumn	142	(24)		29	(33)		45	(25)		22	(21)		
Winter	154	(27)		21	(24)		50	(28)		24	(23)		
Prepregnancy BMI (kg/m^2^)													22.2 ± 0.2 (15–44)
< 18.5	51	(9)		10	(11)		12	(7)		11	(11)		
18.5–25	426	(74)		68	(77)		133	(75)		77	(75)		
25–30	68	(12)		6	(7)		20	(11)		8	(8)		
≥ 30	27	(5)		4	(5)		13	(7)		6	(6)		
Missing	7									1			
Parity													
1	264	(46)		43	(49)		83	(47)		57	(55)		
2	207	(36)		25	(28)		70	(39)		35	(34)		
≥ 3	107	(18)		20	(23)		25	(14)		11	(11)		
Missing	1												
Folic acid intake (3 months before inclusion)	100	(17)		7	(8)		22	(12)		16	(16)		
High blood pressure before/during pregnancy	32	(6)		2	(2)		26	(15)		4	(4)		
Cesarean delivery	100	(17)		14	(16)		42	(24)		10	(10)		
Gestational age at inclusion (weeks)*a*													11.7 ± 0.1 (4–19)
Gestational duration (weeks)*a*													39.3 ± 0.01 (26–42)
Infant characteristics													
Sex													
Girls	280	(48)		35	(42)		91	(51)		22	(21)		
Boys	299	(52)		48	(58)		87	(49)		81	(79)		
Missing				5*b*									
Birth weight (g)													3,376 ± 20 (1,050–4,760)
Birth length (cm)													49.7 ± 0.1 (35–56)
Head circumference at birth (cm)													34.6 ± 0.01 (25–39)
**a**In weeks of amenorrhea. **b**Five medical pregnancy terminations without available data on fetus sex.													

Major congenital anomalies were less frequent for women who conceived during the summer [vs. spring: OR = 0.5; 95% confidence interval (CI), 0.2–1.0] and for those with periconceptual folic acid supplementation (OR = 0.4; 95% CI, 0.2–0.9). Risk factors for FGR were low maternal education level (primary/secondary school vs. baccalaureate: OR = 1.7; 95% CI, 1.1–2.9), smoking (OR = 1.7; 95% CI, 1.1–2.5), or alcohol consumption (≥ 1 drink/day vs. abstinent: OR = 2.5; 95% CI, 0.9–6.9) in early pregnancy, and high blood pressure during pregnancy (OR = 2.9; 95% CI, 1.7–5.1). SHC was less common in the babies of women living in cities (cities vs. rural or semirural/rural areas: OR = 0.5; 95% CI, 0.3–1.0), who were multiparous (≥ 3 vs. 1: OR = 0.5; 95% CI, 0.2–0.9), or who had cesarean versus vaginal deliveries (OR = 0.5; 95% CI, 0.3–1.0), and SHC was more common in boys than girls (OR = 3.4; 95% CI, 2.1–5.7).

*Urinary measurements of atrazine and other herbicides.* The 579 subcohort urine samples included only 10 (2%) with quantifiable atrazine and 24 (4%) with quantifiable atrazine mercapturate (Table 2); the prevalence of direct exposure to atrazine (quantification of at least one of these two compounds) was 6% (32 samples). We observed quantifiable levels of simazine or simazine mercapturate in 8% of the subcohort. We quantified dealkylated triazine metabolites in 20% of subcohort urine samples and hydroxylated triazine metabolites in 40%. We observed the highest urinary concentrations among the dealkylated triazine metabolites. Median and maximum values were lowest for the chloroacetanilide compounds and for atrazine mercapturate.

**Table 2:** Levels of triazine and chloroacetanilide herbicides quantified in urine from 579 randomly selected women from the PELAGIE cohort in early pregnancy (France, 2002–2006).

Table 2. Levels of triazine and chloroacetanilide herbicides quantified in urine from 579 randomly selected women from the PELAGIE cohort in early pregnancy (France, 2002–2006).						
Herbicide		LOQ (μg/L)		*n* (%) > LOQ		Median (95th percentile/maximum) among quantified values
Triazine compounds (μg/L)						
Atrazine		0.05		10 (2)		0.12 (0.52/0.52)
Atrazine mercapturate		0.02		24 (4)		0.05 (0.49/0.68)
Simazine		0.2		6 (1)		1.0 (4.4/4.4)
Simazine mercapturate		0.06		44 (8)		0.5 (3.4/4.6)
Desethylatrazine*a*		0.003		60 (10)		0.1 (7.8/14.0)
Desisopropyl atrazine*a*		0.9		25 (4)		1.4 (7.1/8.2)
2-Chlorodiaminoatrazine*a*		0.5		42 (7)		1.0 (2.9/5.0)
Hydroxyatrazine*b*		0.02		57 (10)		0.1 (1.1/4.0)
Hydroxydesethylatrazine*b*		0.315		31 (5)		0.6 (2.5/2.5)
Hydroxysimazine*b*		0.02		50 (9)		0.08 (1.1/1.6)
Hydroxy desisopropyl atrazine*b*		0.15		90 (16)		0.6 (3.5/4.5)
Ammeline*b*		0.25		79 (14)		0.9 (5.9/7.1)
Chloroacetanilide compounds (μg/L)						
Alachlor		0.006		64 (11)		0.03 (0.21/0.65)
2,6-Diethylaniline		0.01		54 (9)		0.03 (0.13/0.82)
Metolachlor		0.002		144 (25)		0.01 (0.18/1.14)
Acetochlor		0.02		51 (9)		0.05 (0.17/1.04)
Creatinine (mg/L)				579 (100)		1,009 (2,014/3,511)
Sum of compounds (nmol/L)*c*						
Σ(atrazine, atrazine mercapturate)				32 (6)		0.2 (1.4/4.4)
Σ(simazine, simazine mercapturate)				47 (8)		2.0 (12.6/21.8)
Σ dealkylated triazine metabolites				117 (20)		5.3 (34.6/74.8)
Σ hydroxylated triazine metabolites				229 (40)		4.4 (30.9/66.5)
Σ(alachlor, 2,6-diethylaniline)				103 (18)		0.2 (1.0/7.2)
**a**Dealkylated triazine metabolites (desethylatrazine, desisopropyl atrazine, 2-chlorodiaminoatrazine). **b**Hydroxylated triazine metabolites (hydroxyatrazine, hydroxysimazine, hydroxydesethylatrazine, hydroxy desisopropyl atrazine, ammeline). **c**Compounds were summed according to the definition of exposure profiles of interest, as stated in “Materials and Methods.” For example, Σ(atrazine, atrazine mercapturate) means that atrazine or atrazine mercapturate was found in 32 urine samples (sample may contain both compounds) with a median concentration of 0.2 nmol/L.						

*Factors potentially associated with quantification of urinary atrazine or other herbicides.* We observed evidence of direct exposure to atrazine (i.e., quantification of atrazine or atrazine mercapturate) in samples collected during all years of the study (Figure 1). The proportion of samples with evidence of direct exposure did not differ substantially between samples from women living in cities and rural areas, or according to categories of estimated atrazine-contaminated tap water consumption. However, it was higher in samples from women with the highest fish intake. We observed similar conclusions for alachlor exposure.

We quantified simazine or simazine mercapturate, and dealkylated and hydroxylated triazine metabolites more frequently in urine samples of women living in the Côtes d’Armor district than in other municipalities, in rural areas than in cities, and in municipalities with the highest level of atrazine-contaminated tap water. The frequency of the dealkylated metabolites in urine decreased with the proportion of land devoted to corn in the mothers’ home municipality (Figure 1).

We quantified chloroacetanilide compounds more frequently in samples from women residing in Côtes d’Armor than women in other districts. Living in rural areas or near corn crops did not appear to explain the presence of metolachlor in urine samples, but the proportion of samples with evidence of metolachlor exposure was higher in samples from women with the highest intakes of fish and seafood (Figure 1).

In addition, our data showed no correlation between concentrations of triazines and gestational age at urine collection (4–19 weeks) and no relation between the biomarker levels and the time (in days) that samples spent at ambient temperatures (data not shown), suggesting that it is unlikely that these sources of between-subject variation affected our results.

*Association between exposure to atrazine and other herbicides and adverse pregnancy outcomes.* Table 3 describes the herbicide compounds that co-occurred with atrazine exposure in this study. In the 32 urine samples from women in the subcohort with evidence of direct exposure to atrazine, we also quantified alachlor, metolachlor, hydroxyatrazine, acetochlor, simazine, simazine mercapturate, and desisopropyl desethylatrazine and its hydroxylated form more frequently than expected: We found alachlor in 14 samples (44% vs. 10% in the subcohort), metolachlor in 16 samples (50% vs. 25%), and hydroxyatrazine in 10 samples (31% vs. 10%).

**Table 3:** Herbicide compounds co-occurring with atrazine exposure in urine samples of 579 pregnant women (France, 2002–2006).

Table 3. Herbicide compounds co-occurring with atrazine exposure in urine samples of 579 pregnant women (France, 2002–2006).						
		No. of urine samples with the co-occurring compound				
Co-occurring compound		With no exposure to atrazine*a*		With atrazine exposure*b*		With direct atrazine exposure*c*
Total		425		154		32
Atrazine		0		10		10
Atrazine mercapturate		0		24		24
Desethylatrazine		0		60		3
Hydroxyatrazine		0		57		10
Hydroxydesethylatrazine		0		31		3
Simazine		0		6		2
Simazine mercapturate		20		24		5
Hydroxysimazine		20		30		3
Desisopropyl atrazine		20		5		0
2-Chlorodiaminoatrazine		27		15		5
Hydroxy desisopropyl atrazine		68		22		3
Ammeline		46		33		7
Alachlor		35		29		14
2,6-Diethylaniline		39		15		6
Metolachlor		81		63		16
Acetolachlor		34		17		9
**a**Samples with no quantified level of atrazine and its four specific metabolites (desethylatrazine, hydroxyatrazine, hydroxydesethylatrazine, and atrazine mercapturate). **b**Samples with at least one quantified compound among atrazine or its four specific metabolites. **c**Samples with a quantified level of atrazine or atrazine mercapturate.						

Major congenital anomalies were not associated with biomarkers of triazine exposure but were associated with exposure to simazine (OR = 1.8; 95% CI, 1.0–3.5; *p* = 0.07) (Table 4). Atrazine exposure (i.e., quantification of atrazine or one of its specific metabolites) was associated with FGR (OR = 1.5; 95% CI, 1.0–2.2) and SHC (OR = 1.7; 95% CI, 1.0–2.7). Associations with direct exposure to atrazine were also positive but were not statistically significant (Table 4). We found no evidence of an association between exposure to other triazines and FGR or SHC, or between chloroacetanilide herbicides and any of the dichotomous pregnancy outcomes.

**Table 4:** Association between pregnancy outcomes and exposure to herbicides assessed from urinary metabolites quantified in maternal samples collected in early pregnancy, adjusted for co-occurring exposures to other herbicides (France, 2002–2006).

Table 4. Association between pregnancy outcomes and exposure to herbicides assessed from urinary metabolites quantified in maternal samples collected in early pregnancy, adjusted for co-occurring exposures to other herbicides (France, 2002–2006).																
Herbicide exposure defined by grouping metabolites quantified in urine samples			Major congenital anomalies					FGR					SHC			
			*n*		OR*a* (95% CI)			*n*		OR*b* (95% CI)			*n*		OR*c* (95% CI)	
Atrazine and its specific metabolites*d*																
	None		62		Reference			117		Reference			65		Reference	
	At least one		26		1.2	(0.7–2.1)		61		1.5	(1.0–2.2)*e*		38		1.7	(1.0–2.7)*e*
	At least atrazine or atrazine mercapturate (direct exposure)		6		1.0	(0.3–3.1)		14		1.6	(0.8–3.1)		6		1.3	(0.5–3.3)
Simazine and its specific metabolites*f*																
	None		71		Reference			150		Reference			90		Reference	
	At least one		17		1.8	(1.0–3.5)		28		1.1	(0.7–1.8)		13		0.8	(0.4–1.7)
	At least simazine or simazine mercapturate		9		1.6	(0.7–3.8)		16		1.0	(0.5–1.9)		7		0.6	(0.2–1.6)
Dealkylated triazine metabolites*g*																
	No dealkylated metabolite		74		Reference			140		Reference			84		Reference	
	At least one		14		0.9	(0.5–1.7)		38		0.9	(0.6–1.5)		19		0.8	(0.4–1.4)
Hydroxylated triazine metabolites*h*																
	No hydroxylated metabolite		48		Reference			107		Reference			58		Reference	
	At least one		40		1.3	(0.8–2.2)		71		0.8	(0.6–1.3)		45		1.1	(0.7–1.7)
Alachlor																
	None		71		Reference			144		Reference			84		Reference	
	At least alachlor or 2,6-diethylaniline		17		0.9	(0.4–1.7)		34		1.3	(0.8–2.0)		19		0.9	(0.5–1.7)
Metolachlor																
	No		70		Reference			133		Reference			78		Reference	
	Yes		18		0.8	(0.4–1.4)		45		1.2	(0.8–1.9)		25		1.0	(0.6–1.7)
Acetochlor																
	No		82		Reference			169		Reference			94		Reference	
	Yes		6		1.0	(0.4–2.5)		9		0.6	(0.3–1.2)		9		0.8	(0.4–1.6)
Logistic models were adjusted according to backward selection (*p* < 0.20). The exposure of interest was forced in the model (whatever its *p*-value). The retained covariates in final models are listed in the table notes. **a**Retained covariates at *p* < 0.20: year of enrollment, season at conception, mother’s occupational exposure to solvents, gestational age at birth, and simazine exposure (except for the models on dealkylated and hydroxylated metabolites). **b**Retained covariates at *p* < 0.20: smoking status at enrollment, high blood pressure before/during pregnancy, thawing and refreezing process, acetochlor exposure, and atrazine exposure. **c**Retained covariates at *p* < 0.20: residence district, alcohol consumption at enrollment (except for the models on dealkylated and hydroxylated metabolites), thawing and refreezing process, cesarean delivery, parity, and atrazine exposure. **d**Desethylatrazine, hydroxyatrazine, hydroxydesethylatrazine, and atrazine mercapturate. **e**Statistical significance at 5% (the CI does not include 1). **f**Hydroxysimazine and simazine mercapturate. **g**Dealkylated metabolites: desethylatrazine, desisopropyl atrazine, and 2-chlorodiaminoatrazine. **h**Hydroxylated metabolites: hydroxyatrazine, hydroxydesethylatrazine, hydroxysimazine, hydroxy desisopropyl atrazine, and ammeline.																

Birth weight, birth length, and head circumference were all lower in babies of women with atrazine or atrazine mercapturate quantified in their urine than in babies of women not exposed to atrazine; the decrease was statistically significant for birth weight (–151 g; 95% CI, –293 to –9 g) (Table 5). Growth indicators were positively associated with direct exposure to simazine (birth weight: 130 g; 95% CI, 5–255; length: 0.61 cm; 95% CI, 0.1–1.2; head circumference: 0.38 cm; 95% CI, –0.03 to 0.8) and with alachlor or its metabolite in urine (birth weight: 91 g; 95% CI, 3–179).

**Table 5:** Association between intrauterine growth indicators and exposure to herbicides assessed from urinary metabolites quantified in maternal samples collected in early pregnancy, adjusted for co-occurring exposures to other herbicides (France, 2002–2006).

Table 5. Association between intrauterine growth indicators and exposure to herbicides assessed from urinary metabolites quantified in maternal samples collected in early pregnancy, adjusted for co-occurring exposures to other herbicides (France, 2002–2006)															
Herbicide exposure defined by grouping metabolites quantified in urine samples			Subcohort		Birth weight (g)				Birth length (cm)				Head circumference (cm)		
			*n*		β-Coefficient*a* (95% CI)		*p*-Value		β-Coefficient*b* (95% CI)		*p*-Value		β-Coefficient*c* (95% CI)		*p*-Value
Atrazine and its specific metabolites*d*															
	None		425		Reference				Reference				Reference		
	At least one		154		–57	(–130 to 17)	0.13		–0.22	(–0.5 to 0.1)	0.20		–0.18	(–0.4 to 0.1)	0.16
	At least atrazine or atrazine mercapturate (direct exposure)		32		–151	(–293 to –9)	0.04		–0.44	(–1.1 to 0.2)	0.19		–0.39	(–0.9 to 0.1)	0.10
Simazine and its specific metabolites*e*															
	None		506		Reference				Reference				Reference		
	At least one		73		40	(–63 to 142)	0.45		0.17	(–0.3 to 0.6)	0.48		0.27	(–0.1 to 0.6)	0.11
	At least simazine or simazine mercapturate		47		130	(5 to 255)	0.04		0.61	(0.1 to 1.2)	0.03		0.38	(–0.03 to 0.8)	0.07
Dealkylated triazine metabolites*f*															
	No dealkylated metabolite		462		Reference				Reference				Reference		
	At least one		117		–18	(–99 to 62)	0.65		0.03	(–0.3 to 0.4)	0.87		0.18	(–0.1 to 0.4)	0.18
Hydroxylated triazine metabolites*g*															
	No hydroxylated metabolite		350		Reference				Reference				Reference		
	At least one		229		–12	(–79 to 55)	0.72		0.002	(–0.3 to 0.3)	0.99		–0.09	(–0.3 to 0.1)	0.41
Alachlor															
	None		476		Reference				Reference				Reference		
	At least alachlor or 2,6–diethylaniline		103		91	(3 to 179)	0.04		0.35	(–0.05 to 0.7)	0.08		0.11	(–0.2 to 0.4)	0.45
Metolachlor															
	No		435		Reference				Reference				Reference		
	Yes		144		5	(–78 to 87)	0.91		–0.20	(–0.6 to 0.1)	0.25		0.04	(–0.2 to 0.3)	0.73
Acetochlor															
	No		528		Reference				Reference				Reference		
	Yes		51		65	(–53 to 183)	0.28		0.13	(–0.4 to 0.7)	0.63		0.09	(–0.3 to 0.5)	0.65
Linear models were adjusted according to backward selection (*p* < 0.20). The exposure of interest was forced in the model (whatever its *p*-value). The retained covariates in final models are listed in the table notes. **a**Retained covariates: year of enrollment, education level, smoking status at enrollment, high blood pressure before/during pregnancy, thawing and refreezing process, prepregnancy BMI, child’s sex, shellfish intake (except for the models on dealkylated and hydroxylated metabolites), gestational age at birth, squared gestational age, alachlor exposure, and atrazine exposure. **b**Retained covariates: year of enrollment, smoking status at enrollment, high blood pressure before/during pregnancy, season at conception, prepregnancy BMI, child’s sex, shellfish intake, gestational age at birth, squared gestational age, alachlor exposure, and atrazine exposure (except for the models on dealkylated and hydroxylated metabolites and on metolachlor). **c**Retained covariates: residence district, smoking status at enrollment, prepregnancy BMI, child’s sex, cesarean delivery, parity, shellfish intake, gestational age at birth, squared gestational age, atrazine exposure, and simazine exposure. **d**Desethylatrazine, hydroxyatrazine, hydroxydesethylatrazine, and atrazine mercapturate. **e**Hydroxysimazine and simazine mercapturate. **f**Dealkylated metabolites: desethylatrazine, desisopropyl atrazine, and 2-chlorodiaminoatrazine. **g**Hydroxylated metabolites: hydroxyatrazine, hydroxydesethylatrazine, hydroxysimazine, hydroxy desisopropyl atrazine, and ammeline.															

Analyses of the babies of the 144 women with quantifiable metolachlor indicated a significant decrease in head circumference (for a 1-nmol/L increase in metolachlor, β = –0.53 cm; 95% CI –0.96 to –0.10) and nonsignificant decreases in birth weight (–21 g; 95% CI, –151 to 109) and birth length (–0.24 cm; 95% CI, –0.78 to 0.31).

Additional analyses showed a weak positive association between quantification of atrazine or one of its specific metabolites in urine and male genital anomalies (OR = 1.4; 95% CI, 0.6–3.2; based on 5 cases classified as exposed and 18 as unexposed) and a stronger but nonsignificant association with evidence of direct atrazine exposure (OR = 2.3; 95% CI, 0.6–8.4; based on 3 cases classified as exposed and 18 as unexposed) compared with no exposure. Among the 35 mothers with quantified atrazine or atrazine mercapturate in their urine, the OR for male genital anomalies associated with a 1-nmol/L increase in these compounds was 2.8 (95% CI, 0.9–8.6).

## Discussion

This study showed the presence of atrazine residues in urine samples of pregnant women up to 3 years after atrazine was banned in Europe. Only a few studies have measured urinary levels of triazine compounds in general and nonfarm populations (Bakke et al. 2009; Curwin et al. 2007), mainly in the United States; none focused on pregnant women. Curwin et al. (2007) studied 51 nonfarm children in Iowa in 2001 and observed higher levels of atrazine mercapturate than in our study: 12% had detectable levels (and their LOD at 1.16 μg/L was higher), and the maximum level was 2.2 μg/L. Another Iowa study of 10 nonfarm men in 2002–2003 observed urinary traces of atrazine mercapturate similar to our study (6% of detectable levels, with an LOD < 0.2 μg/L) (Bakke et al. 2009). As Barr et al. (2007) suggested and our results confirm, measurement of atrazine mercapturate alone misrepresents and underestimates total atrazine exposure. We measured urinary concentrations of other triazine metabolites that may also be environmental degradates and found higher detection frequencies and levels for most of them than for atrazine or atrazine mercapturate. That metabolite traces are more frequent than the parent substance has also been generally observed in environmental media. The detection of atrazine and simazine residues in urine samples of pregnant women up to 3 years after they were banned in Europe in 2003 suggests that these pesticides persist several years in the environment. However, we cannot rule out the possibility of uses on crops of forbidden products that represented 6% of the used products in 2006 in Brittany (Agreste Bretagne 2009). In the present study, we found no evidence of a link between herbicide exposure biomarkers and area devoted to corn production in the mother’s municipality of residence. Although our assessment of atrazine contamination in tap water was crude and limited to the Ille-et-Vilaine district and to a period earlier than the study period, our descriptive results suggest that water contamination was a likely determinant of urinary levels of triazine compounds in our Britton population. Curwin et al. (2007) found high correlations between the atrazine mercapturate urinary levels of members of the same family in nonfarm families and suggested possible common sources of exposure for them, such as diet or water after environmental atrazine contamination.

Our study suggests that environmental atrazine exposure may impair fetal growth, as shown by outcomes such as FGR and SHC that may be considered as pathological entities. This result was reinforced by inverse associations between atrazine exposure and the crude fetal growth indicators, especially birth weight. To our knowledge, until now only limited animal data have suggested that gestational atrazine exposure may affect fetal growth. Although Rayner et al. (2004) showed a decrease in rat pup weight after *in utero* exposure to atrazine alone, the same team observed the opposite effect when dams were exposed to a low-level mixture of atrazine and four degradates (Enoch et al. 2007). Little is known about the potential developmental toxicity of either the dealkylated or hydroxylated forms of triazine compounds. Other studies have suggested that dealkylated atrazine metabolites that retain the chlorine molecule may be as potent as the parent substance, whereas hydroxylated metabolites have lower potency (Laws et al. 2003; Stoker et al. 2002). Our study suggests that any adverse effect of these dealkylated and hydroxylated compounds on fetal growth outcomes would likely be driven mainly by metabolites specific to atrazine (desethylatrazine, hydroxyatrazine, or hydroxydesethylatrazine) and not by other dealkylated or hydroxylated triazine metabolites. Finally, our results are consistent with those of Munger et al. (1997), Villanueva et al. (2005), and Ochoa-Acuña et al. (2009), all of whom examined exposure during pregnancy to atrazine and its degradates by consumption of tap water. We did not, however, confirm associations between major congenital anomalies and atrazine exposure suggested in several recent ecological studies (Davis 2005; Mattix et al. 2007;Waller et al. 2010; Winchester et al. 2009). A meta-analysis has already linked hypospadias to parental pesticide exposure, with possible endocrine-mediated effects (Rocheleau et al. 2009). We found only one mammal experiment that observed hypospadias in rats treated with atrazine (Wu et al. 2007). Our finding of an association between male genital anomalies and atrazine or atrazine mercapturate levels in early-pregnancy urine samples was based on small numbers, and it requires replication. It would reinforce the hypothesis of endocrine-mediated effects of low levels of atrazine exposure, formulated from animal observations in laboratories and in the wild (Hayes et al. 2006).

Our study suggests, for the first time, to our knowledge, that simazine exposure and alachlor exposure may be positively associated with fetal growth indicators. However, these results need to be replicated. Finally, our study suggests that metolachlor exposure levels may be inversely correlated with head circumference, consistent with the recent report by Barr et al. (2010) of an inverse correlation with birth weight. In the present study, we took into account coexposures to herbicides as potential confounding factors in all models.

Because cohort members were asked to provide only one urine sample for this study, possible intraindividual variability cannot be considered. We assumed that one sample was enough to reflect chronic exposure resulting from environmental atrazine contamination. Chemical stability analyses suggest that our study may have nondifferentially underestimated atrazine exposure levels, because of routine post shipment of urine samples.

## Conclusion

This is the first study to assess the association between urinary biomarkers of exposure to triazine compounds during pregnancy and adverse birth outcomes and to take into account exposure to mixtures of atrazine, its environmental degradates, and other co-occurring herbicides. Because atrazine is banned in Europe, this study deals with environmental contamination by atrazine and its persistence in environment, which leads to low levels of atrazine exposure. Our results thus raise particular concerns for countries where atrazine is still in use.
